# Supplementary material for: Vaginal and Uterine Microbiota of Healthy Maiden Mares during Estrus
Source: Vet Sci. 2024 Jul 18;11(7):323. doi: 10.3390/vetsci11070323 (PMC11281598; doi:10.3390/vetsci11070323)
Supplement: Supplementary file 1 [file vetsci-11-00323-s001.zip › vetsci-3075101-supplementary.pdf]

Supplementary Table S1. PERMANOVA results on Jaccard and Bray-Curtis distances on sample ASV composition of uterine, vaginal, clitoral fossa (CF) and perineal skin samples from 12 healthy mares during estrus.

**Distance metric**	**Pseudo-*F* statistic**	**R^2**	***P* value**
Jaccard	1.96	0.140	0.001
Bray-Curtis	2.75	0.187	0.001
